# Thrombotic Thrombocytopenic Purpura Without Evidence of Microangiopathic Hemolytic Anemia: A Case Report

**DOI:** 10.7759/cureus.35927

**Published:** 2023-03-09

**Authors:** Rachaita Lakra, James Lopez, Christopher Graham

**Affiliations:** 1 Internal Medicine, Louisiana State University Health Sciences Center, Shreveport, USA; 2 Hematology and Medical Oncology, Louisiana State University Health Sciences Center, Shreveport, USA; 3 Hematology and Oncology, Louisiana State University Health Sciences Center, Shreveport, USA

**Keywords:** caplacizumab, plasmapheresis, adamts13, microangiopathic hemolytic anemia, thrombotic thrombocytopenic purpura

## Abstract

Diagnosis of thrombotic thrombocytopenic purpura (TTP) is challenging due to varied clinical presentations and is primarily based on ADAMTS13 activity assay, however clinical suspicion to include TTP as a potential diagnosis relies on multiple scoring systems all involving hemolysis as a prime feature. Here, we report a case of TTP without any evidence of microangiopathic hemolytic anemia (MAHA). A 65-year-old male admitted with a Glasglow come scale of 3 was intubated and sedated on admission. Complete blood count was concerning for a hemoglobin (Hb) of 5.8 g/dL, and a platelet count of 76 k/µL. The patient had a bleeding episode while placing a central line; the repeat platelet count was found to be 35 k/µL, further dropping to 14 k/µL the next day. Coagulation studies now reflected PT of 19.8 sec, aPTT of 38.7 sec, and fibrinogen of 212 mg/dL. The peripheral smear showed no evidence of hemolysis. TTP was kept low on the differential and haematological anomalies were attributed to possible disseminated intravascular coagulation (DIC) from sepsis and liver disease. ADAMSTS13 was incidentally checked upon admission, later resulting in <5% activity with a Bethesda titer inhibitor of 3.2. The patient was immediately initiated on plasmapheresis along with prednisone. Additionally, rituximab and caplacizumab were added. Plasmapheresis was continued for ten sessions until the platelet count reached 167 k/µL. At the time of discharge, laboratory values revealed platelets of 251 k/µL and hemoglobin of 8.8 g/dL. We recognize that the diagnosis of TTP is challenging because of its diverse clinical manifestations and constrained availability of ADAMTS13 testing. Clinical prediction scores have been developed to estimate the pretest probability of severe ADAMTS13 deficiency, however, they all include the presence of MAHA. Atypical presentation of TTP has been previously acknowledged however continues to remain under-recognized.

## Introduction

Thrombotic thrombocytopenic purpura (TTP), a subtype of thrombotic microangiopathy was first described in the year 1925 by Moschowitz [1]. TTP was initially described as a pentad is only seen in 10% of patients and has now evolved to accept other manifestations of TTP [2]. TTP remains well established as a rare hematological emergency with an average annual prevalence of approximately 10 cases/million and an annual incidence of one new case/million people [3]. The pathogenesis of TTP includes the decreased activity of plasma metalloproteinase ADAMTS13 (a disintegrin and metalloproteinase with a thrombospondin type 1 motif, member 13), which is an important enzyme involved in the cleavage of von Willebrand factor (VWF) multimers [4]. ADAMTS13, located on chromosome 9q34, encodes a multidomain protein of 1427 amino acids [5]. Without cleavage, these large multimers accumulate microthrombi leading to hemolytic anemia, thrombocytopenia, and further diverse thrombotic complications causing multiorgan failure [6]. 

Diagnosis of TTP is challenging due to its varied clinical presentations and is primarily based on ADAMTS13 activity assay, however, clinical suspicion to include TTP as a potential diagnosis relies on multiple scoring systems all involving hemolysis as a prime feature. We report an unusual case of TTP without any evidence of microangiopathic hemolytic anemia (MAHA).

## Case presentation

A 65-year-old male was presented to the emergency department by paramedics with a Glasglow come scale of 3; the patient was intubated and sedated on admission. The patient remained hypotensive with a blood pressure of 68/50 mmHg requiring vasopressor support and transfer to the ICU. Admission complete blood count was concerning for hemoglobin of 5.8 g/dL, and platelet count of 76 k/µL (Table 1). Anemia workup included ferritin of 11,141 ng/mL, iron saturation of 123%, high normal vitamin B12 of 1140 pg/mL, and folate of 1.9 ng/mL. The chemistry panel showed a creatinine of 3.20 mg/dL, an aspartate aminotransferase (AST) of 223 U/L, alanine transaminase (ALT) of 66 U/L, and total bilirubin of 6.9 mg/dL (Table 2).

**Table 1:** Laboratory results of the case before and after plasmapheresis PT: prothrombin time; aPTT: partial thromboplastin time; ADAMTS13: a disintegrin-like metalloprotease domain with thrombospondin type 1 motifs

Laboratory results (Units of measurement)	Reference Values	On Admission	Post Plasmapheresis
Hemoglobin (g/dL)	12.5-16.3 g/dL	5.6	8.8
Platelet Count (k/µL)	150-450 k/µL	76	251
Creatinine (mg/dL)	0.50-1.40 mg/dL	3.20	0.70
PT (sec)	9.4-12.5 sec	19.8	12.9
aPTT (sec)	25.1-36.5 sec	44.8	28.3
ADAMTS13 activity (%)	>/= 70 %	< 5	70

**Table 2:** Other laboratory results of the case AST: aspartate aminotransferase; ALT: alanine transaminase

Laboratory results (Units of measurement)	Reference Values	Patient’s Results
Iron saturation (%)	20-50 %	123
Ferritin (ng/mL)	20-300 ng/mL	11,141
Folate (ng/mL)	4-24 ng/mL	1.9
Vitamin B12 (pg/mL)	210-950 pg/mL	1140
AST (U/L)	10-40 U/L	223
ALT (U/L)	10-44 U/L	66
Total Bilirubin (mg/dL)	0.1-1 mg/dL	6.9
Haptoglobin (mg/dL)	30-250 mg/dL	65
Lactic Dehydrogenase (IU/L)	105-333	334
Fibrinogen (mg/dL)	214-454 mg/dL	212
ADAMTS 13 Bethesda Inhibitor Titer	<0.4	3.2

The hepatitis panel and HIV were negative. Coagulation studies such as prothrombin time/international normalized ratio (PT/INR), partial thromboplastin time (aPTT), and haptoglobin were normal. lactate dehydrogenase (LDH) was slightly elevated at 334 U/L. The peripheral smear showed no evidence of hemolysis. The patient received two units of packed red blood cells, increasing the hemoglobin to 7.1 g/dL. The patient had a bleeding episode while placing a central line, subsiding with aggressive pressure. Repeat platelet count was found to be 35 k/µL, further dropping to 14 k/µL the next day. Coagulation studies now reflected PT of 19.8 sec, aPTT of 38.7 sec, and fibrinogen of 212 mg/dL. Hematology was consulted for the probability of disseminated intravascular coagulation (DIC). Since there were no concerns for hemolysis on the smear, TTP was kept low on the differential and hematological anomalies were attributed to possible DIC from sepsis and liver disease. Immature platelet function was 6.1% which is normal despite thrombocytopenia, indicating bone marrow suppression. The patient was therefore scheduled for a bone marrow biopsy. ADAMSTS13 was incidentally checked upon admission, later resulting as <5% (normal range 50%-160%, confirmatory level < 10%) activity with a Bethesda titer inhibitor of 3.2.

The patient was diagnosed with a rare occurrence of TTP without hemolysis and immediately initiated plasmapheresis along with dexamethasone 40 mg daily for four days, it was later switched to prednisone 1 mg/kg/day, and bone marrow biopsy was deferred. He was also initiated on rituximab 375 mg/m2 weekly for four doses due to the presence of the inhibitor. He briefly received caplacizumab which was discontinued due to bleeding. Plasmapheresis was continued for ten sessions until the platelet count reached 167 k/µL. At the time of discharge, laboratory values revealed platelets of 251 k/µL and hemoglobin of 8.8 g/dL (Table 1). Despite this, the patient had a prolonged hospitalization for multiple issues including Methicillin-resistant Staphylococcus aureus (MRSA) endocarditis and chronic respiratory failure requiring tracheostomy placement eventually requiring long-term assisted facility placement. 

## Discussion

Even though the patient presented with anemia, thrombocytopenia, renal dysfunction, and neurological deficits, the diagnosis of TTP was made on the basis of ADAMTS13 <5% since the blood smear lacked pathognomonic schistocytes and evidence of hemolysis. The diagnosis of acquired TTP can also be missed in some instances if the inhibitor levels are too low to be detected in the conventional mixing test for inhibitors [7].

Atypical TTP has been acknowledged previously, around twenty years ago [8], and has been mentioned in textbooks as well [9], however, we continue to base our diagnosis on the pentad and dyad (MAHA and thrombocytopenia) presentation. Neurological manifestations of TTP are repeatedly misdiagnosed as acute stroke. A case series reported ten patients with completely varied clinical presentations of TTP [10]. Downes et al. described two women with only neurological presentation and without any hematological anomalies [11]. Patients with TTP receiving tissue plasminogen activator (tPA) or unnecessary procedures can cause catastrophic outcomes. Badugu et al. reported a case of a patient admitted for slurring of speech who received tPA as per stroke protocol. This patient was later found to have TTP [12].

Timely initiation of plasmapheresis is a lifesaving intervention in patients with TTP which reduces the mortality rate from 90% to approximately 20% [13]. Our patient responded well to plasmapheresis with a final increase of platelet count from 35 k/µL to 251 k/µL in ten sessions. Additional therapy apart from plasmapheresis and steroids has been used for the management of TTP including rituximab which induces remission for more than 19 months [14] and caplacizumab which causes suppression of VWF for 48 hours and reduces overall relapse rates [15]. We included both rituximab and caplacizumab in our managerial approach. 

We recognize that the diagnosis of TTP is challenging because of its diverse clinical manifestations and constrained availability of ADAMTS13 testing. Clinical prediction scores have been developed to estimate the pretest probability of severe ADAMTS13 deficiency; however, they all include the presence of MAHA. Atypical presentation of TTP has been previously acknowledged, however, it continues to remain under-recognized. 

## Conclusions

This case report serves as a reminder of atypical TTP and emphasizes considering TTP as the potential diagnosis despite the absence of hemolysis. TTP is recognized as a dire hematological emergency and emergent initiation of therapeutic plasma exchange is lifesaving. 
